# Demographic, Operational, and Healthcare Utilization Factors Associated with Emergency Department Patient Satisfaction

**DOI:** 10.5811/westjem.2015.4.25074

**Published:** 2015-06-22

**Authors:** Matthew W. Morgan, Joshua G. Salzman, Robert C. LeFevere, Avis J. Thomas, Kurt M. Isenberger

**Affiliations:** * Regions Hospital, Department of Emergency Medicine, St. Paul, Minnesota; † HealthPartners Institute for Education and Research, Minneapolis, Minnesota

## Abstract

**Introduction:**

The primary aim of this study was to determine which objectively-measured patient demographics, emergency department (ED) operational characteristics, and healthcare utilization frequencies (care factors) were associated with patient satisfaction ratings obtained from phone surveys conducted by a third-party vendor for patients discharged from our ED.

**Methods:**

This is a retrospective, observational analysis of data obtained between September 2011 and August 2012 from all English- and Spanish-speaking patients discharged from our ED who were contacted by a third-party patient satisfaction vendor to complete a standardized nine-item telephone survey by a trained phone surveyor. We linked data from completed surveys to the patient’s electronic medical record to abstract additional demographic, ED operational, and healthcare utilization data. We used univariate ordinal logistic regression, followed by two multivariate models, to identify significant predictors of patient satisfaction.

**Results:**

We included 20,940 patients for analysis. The overall patient satisfaction ratings were as follows: 1=471 (2%); 2=558 (3%); 3=2,014 (10%), 4=5,347 (26%); 5=12,550 (60%). Factors associated with higher satisfaction included race/ethnicity (Non-Hispanic Black; Hispanic patients), age (patients ≥65), insurance (Medicare), mode of arrival (arrived by bus or on foot), and having a medication ordered in the ED. Patients who felt their medical condition did not improve, those treated in our ED behavioral health area, and those experiencing longer wait times had reduced satisfaction.

**Conclusion:**

These findings provide a basis for development and evaluation of targeted interventions that could be used to improve patient satisfaction in our ED.

## INTRODUCTION

### Background

Recent healthcare reform efforts have increasingly focused on the concept of patient-centered care, which expects patients to actively participate in healthcare decision making and for care to be as individualized as possible.^1^ Patient satisfaction is a metric that has been used to measure healthcare providers’ effectiveness around achieving true patient-centered care. Since 2008, the Hospital Consumer Assessment of Healthcare Providers Survey (HCAHPS) has been administered to patients as a standardized tool to assess patient satisfaction nationwide.^2^ Aggregated results are publicly available online through the Department of Health and Human Services, giving consumers the ability to compare patient satisfaction scores among healthcare providers. Patient satisfaction metrics are also becoming increasingly important financially. The Patient Protection and Affordable Care Act of 2010 (P.L. 111–148) includes HCAHPS among the measures to be used to calculate value-based incentive payments in the hospital Value-Based Purchasing program, beginning with discharges in October 2012.^3^

### Importance

Although the association between patient satisfaction and clinical quality and outcomes has been studied in other care settings, little is known about factors associated with higher patient satisfaction, effective methods for improving satisfaction and what effect satisfaction has on health care outcomes for emergency department (ED) patients.^4^–^6^ Previous studies have identified timeliness of care, provision of information, staff empathy/attitude, and pain management as service factors influencing ED patient satisfaction.^7^–^8^ Demographic factors have been variably associated with satisfaction.^8^–^9^ Non-emergency ambulatory setting patient satisfaction has been correlated with improved patient outcomes, including higher medical compliance, decreased utilization of medical services, less malpractice litigation, and greater willingness to return.^7^,^10^ Following the ED patient satisfaction research agenda proposed by Boudreaux and O’Hea, we focused our attention in this analysis on factors that influenced patient satisfaction using a robust methodology that included multiple demographic, operational, and healthcare utilization variables within a large set of patients.^11^

### Objectives of This Investigation

The primary aim of our study was to determine which objectively measured factors related to patient demographics, ED operational characteristics, and healthcare utilization frequencies (care factors) were associated with patient satisfaction ratings obtained from phone surveys conducted by a third-party vendor for patients discharged from our ED.

## METHODS

### Study Design and Setting

This was a retrospective, observational convenience sample study with the primary aim of assessing objectively measured patient demographic, ED operational, and healthcare utilization factors as predictors of patient satisfaction. Our institution is an urban, upper Midwest Level I Adult and Pediatric Trauma Center with an ED residency training program with approximately 78,000 patient encounters per year. This study was reviewed and approved by the HealthPartners Institutional Review Board, with the consent requirement waived.

### Selection of Participants

Since March 2011, all English- and Spanish-speaking patients discharged from our ED are contacted by a third-party vendor (Emergency Excellence, Brentwood, TN) to complete a standardized nine-item telephone survey by a trained phone surveyor. Patients are contacted up to four times, in the 48-hour period following discharge, before they are determined to be unreachable. In September 2011, the survey methodology changed to a 1–5 scoring system for the satisfaction rating questions (5=best; 1=worst). We included all patients discharged from our ED between September 1, 2011, and August 31st, 2012 who provided a response to the “overall satisfaction” question on the satisfaction survey and associated link to the electronic medical record of the patient’s encounter. Exclusion criteria included patients who were admitted following their ED encounter, patient seen outside of the one-year study period, patients who were unable to be reached to complete the telephone survey, and patients excluded due to proactively placing their name on our institutional research opt-out list.

### Intervention

Using the 1–5 scoring scale, all patients were asked to assess their medical condition compared to the day of their visit, their understanding of the discharge instructions they received, their confidence and trust level in the physicians who treated them, and satisfaction ratings for their overall experience, their physician, their nurse, and their advanced practice provider. The phone survey took approximately 15 minutes to complete. If the patient expressed concern about their care, the phone surveyor relayed the information to the ED quality committee for further follow up.

### Methods and Measurements

Programming staff from our institution electronically abstracted patient demographic information (age, sex, primary language, and zip code of residence) for all patients discharged from the ED between September 1, 2011, and August 31, 2012. Patients with satisfaction scores obtained by the survey vendor had additional demographic, ED operational and healthcare utilization variables abstracted from the electronic medical record and the satisfaction survey database. Additional demographic factors included self-reported race/ethnicity, use of an interpreter in the ED, language spoken during the survey, zip code-based median income, zip code-based percent in poverty, mode of arrival, and payer type. ED operational variables included the day of the week, weekend vs. weekday, calendar month and quarter, time of day (11pm–7am vs. all other times), wait time from arrival to physician and from physician to disposition, and treatment location within the ED. Healthcare utilization variables included whether any medications were ordered in the ED, whether any imaging was performed, whether laboratory tests were performed. We also abstracted for analysis the patient’s self-assessed change in medical condition. The final data set was reviewed for accuracy with the study investigators, and a de-identified analysis database was provided for analysis.

### Outcome

The outcome measure for this analysis was a predicted one-unit increase in patient satisfaction score.

### Data Analysis

We compared age, sex, primary language, and poverty rate based on zip code between the satisfaction survey responders and non-responders to assess potential sampling bias. All potential analysis variables were then tabulated by satisfaction score and reviewed manually (SAS 9.2 and 9.3; Cary, NC). We condensed categorical variables with many possible values into a smaller number of categorical levels before further analysis.

We analyzed the impact of multiple visits within the study period on satisfaction score. A more in-depth analysis of language was also conducted to determine the effect of the listed primary language, use of an interpreter in the ED, and language spoken during the telephone interview on satisfaction score.

Variables were considered for further analysis and inclusion in the regression model if the variable level had a material interaction with the distribution of satisfaction scores. We used ordinal logistic regression models with one predictor in each model to provide preliminary assessments of many possible predictive variables, with the outcome measure equal to a one-unit increase in satisfaction score. Variables with non-significant findings (p>0.10) in univariate analyses or variables with no material difference determined by the investigators were removed from further analyses. We examined relationships between closely related categorical predictors and consolidated strongly overlapping categories to improve the stability of possible multivariate models. A multivariate model was constructed using all remaining predictors, and backwards elimination based on p-values was used to identify the remaining significant predictors. To verify software-generated backward elimination methodology, we also manually completed backwards elimination to monitor developing patterns. Categories of multi-level variables were combined if they were not associated with materially different odds ratios. We then re-checked the model by examining changes to Akaike Information Criterion when excluded variables were added back in. Finally, given that some compensation schemes use a dichotomous satisfaction score we performed a secondary analysis, using satisfaction score=5 vs not 5.

## RESULTS

**Figure 1:**
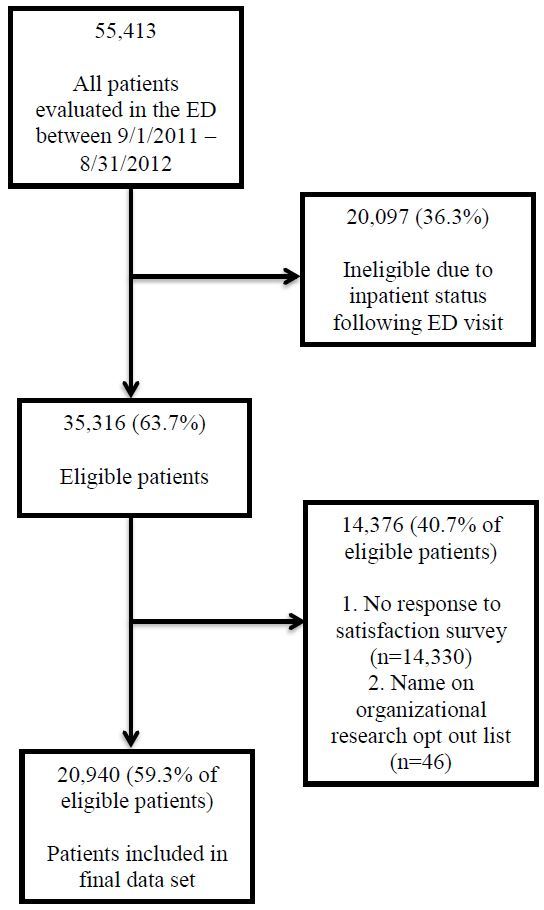
Patient selection flowchart. *ED,* emergency department

Figure 1 shows our patient selection flowchart, with 20,940 (59%) patients included in the final analysis. There were no material differences between responders and non-responders with respect to age, poverty rate based on zip code of residence, language, or chief complaint. There were 10,503 responders to the telephone survey with more than one encounter within the study period. Due to no association with a change-in-satisfaction score based on the number of encounters (OR=0.99; 95% CI [0.97–1.01]), a single visit per patient was randomly selected and included in the analysis.

The overall patient satisfaction ratings were as follows: 1=471 (2%); 2=558 (3%); 3=2,014 (10%), 4=5,347 (26%); 5=12,550 (60%) (Table 1). Use of an interpreter in the ED, zip code-based median income, day of the week, calendar month and quarter, imaging done (yes or no), and lab work done (yes or no) were removed from further analysis after descriptive summaries showed no material differences in satisfaction rates. Variables with statistically significant predictive power from the univariate analyses are seen in Table 2. The only variable excluded from the multivariate analysis was day of the week (OR=1.03; 95% CI [0.97–1.09]). To reduce collinearity in multivariate models, we collapsed age and Medicare status into a single multi-level categorical variable : ≥65 years old; <65 years old and on Medicare; <65 years old and not on Medicare., Similarly, race and language were also consolidated into a single four-level variable: non-Hispanic Black, English-speaking Hispanic, Spanish-speaking Hispanic, all other. We reduced categories for mode of transportation to bus/walk vs. all other because the other modes of transportation (private, ambulance and other) were not significantly different from each other.

**Figure 2:**
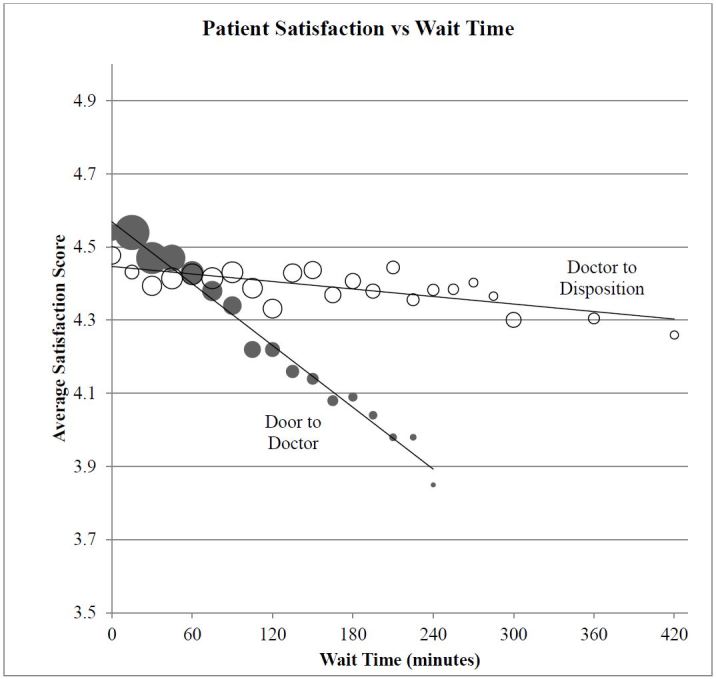
Patient satisfaction scores by wait time (minutes). Size of circle is proportional to sample size of patient with that waiting time.

Accounting for all potentially significant factors included in this analysis, minorities (Black and Hispanic patients), seniors (≥65 years old), patients who arrived by bus or on foot, and those who had a medication ordered in the ED were more likely to have significantly higher overall satisfaction scores (Table 3). Patients who felt their medical condition did not improve or who were treated in our behavioral health unit were less satisfied. Longer wait times, particularly from door to doctor, also reduced patient satisfaction. Figure 2 shows the nearly linear relationship of patient satisfaction across a broad range of wait times. A two-hour increase in the door-to-doctor wait time decreased the five-point patient satisfaction score by an average of 0.34 points. In contrast, a two-hour increase in doctor-to-disposition wait times decreased the patient satisfaction score by only 0.04 points.

We validated the model by examining changes in the model fit (AIC) when excluded variables were added back in one by one. Adding the percent of households below the poverty level improved the model fit; however, the variable itself did not have a material odds ratio and did not materially affect other estimates [OR estimated as 0.99 (95% CI [0.99–1.02], p=0.44) for a 10% change in zip code-based poverty rate]. The number of visits per patient, weekend, nighttime, and more detailed analyses of mode of arrival, age and payer did not improve the model fit and could clearly be discarded.

When patient satisfaction was dichotomized (5 vs. not 5), the only notable difference from the results presented in Table 3 was the magnitude of association between patients <65 and on Medicaid with patient satisfaction (OR=1.08; 95% CI [1.01–1.15]). No other findings were materially different from the ordinal multiple regression model results.

## DISCUSSION

The decision to tie patient satisfaction to provider and hospital reimbursement is a source of passionate debate among ED physicians, hospital administrators, and payors. Given the likelihood that this methodology will continue into the future, identifying factors associated with lower patient satisfaction and design interventions targeted to those variables will be an important process for all care providers to undertake.

### Demographic Factors

The influence of demographic factors on patient satisfaction has not been consistently demonstrated in previous studies. Patient demographics are not modifiable factors for ED providers. However, identifying whether and why differences exist locally are key questions. In our study, patients with Hispanic ethnicity and Spanish as their first language reported higher satisfaction than other patients. This is consistent with previously reported literature.^7^,^8^ In past studies, African Americans have reported similar or lower rates of satisfaction than Caucasians, which was not the case in this investigation.^7^,^12^ For non-English speaking patients, Garret et al. speculated about the presence of a “happy migrant effect,” theorizing that those of another culture and speaking a different language may discount or minimize negative attributes of their care for various reasons, including language difficulties.^13^ The use of interpreters is essentially universal in our ED for those whose primary language is not English, and the patient satisfaction survey was administered in Spanish for non-English speaking Hispanic patients. Identification of the root cause for higher satisfaction in these populations will need to be undertaken through further study, most likely through qualitative research methods.

We also found older patients (≥65 years old) were more likely to be satisfied. Past studies examining age as a factor in patient satisfaction have shown mixed results. Sun found younger patients to be less satisfied, while Boudreaux found older patients more likely to recommend but not more satisfied.^7^,^12^ Others have found no association between age and satisfaction.^14^,^15^ In our patient population, those with Medicare who were less than age 65 and those without insurance were also more likely to report a high level of satisfaction. Other studies have found little relationship between insurance status and satisfaction.^12^,^14^,^15^

### Healthcare Utilization Factors

A previous study examining patient expectations about diagnostic studies found no correlation with overall satisfaction.^16^ In our study, performance of laboratory testing and imaging similarly showed no effect. These results counter conventional wisdom and other published literature that suggests testing makes patients happier.^17^,^18^ The current study did find a positive association between the presence of one or more medication orders and satisfaction score. Previous studies have looked specifically at pain management as a subjective measure and found a positive association with satisfaction.^19^,^20^ Our study did not look at type of medication ordered, so we are unable to discern if patients administered pain medication in our sample reported higher or lower satisfaction than patients receiving other forms of medications. In a recent study by Schwartz et al., there appeared to be no difference in patient satisfaction score (Press-Ganey) and administration of analgesic medication.^21^ Our study found that patients who were feeling “the same” or “worse” at the time of their callback were less satisfied. It may be that managing expectations about when patients will feel better could lead to improved satisfaction.

### Operational Factors

Of the operational factors studied, it is not surprising that the door-to-doctor wait time was one of the strongest predictors of satisfaction (Figure 2). This is in agreement with many previous studies, although others have suggested that the perception of wait time is what is important.^16^,^22^–^24^ Interestingly, the linear relationship between wait time and satisfaction persists over the entire wait-time spectrum, and for two different kinds of wait time (door to doctor; doctor to disposition). The lack of thresholds may indicate that patients do not arrive with clear expectations regarding wait times. The regression line for door-to-doctor wait times as a predictor of patient satisfaction is much steeper than the regression line for doctor-to-disposition times (reduction of 0.34 points for a two hours door-to-doctor wait time vs. 0.04 for door to disposition). This lends credence to the “virtual room” approach from the perspective of patient satisfaction. When using virtual rooms, patients are evaluated by a physician immediately and a treatment plan is initiated. Patients then wait for test results in the waiting room. Additionally, we noted a significantly lower reported satisfaction among patients treated in the mental health area of our ED. This population has been infrequently studied and may have needs that are not well met in a traditional ED setting. The current version of HCAHPS specifically excludes patients discharged with a primary psychiatric diagnosis from the sampling pool.^2^ O’Regan and Ryan did find a generally low level of satisfaction albeit with fewer patients (n=55).^25^ These results raise questions about the effect patient dissatisfaction may have on psychiatric outcomes. Factors that did not impact satisfaction included the day of the week the patient was seen, weekday vs. weekend, month and quarter of the year, as well as the time of day.

## LIMITATIONS

There are limitations to our research design that are important to highlight. The first potential limitation is the use of a telephone survey method for obtaining patient satisfaction results. This methodology relies on patients having an active telephone number and a willingness to complete a survey when contacted. However, responders and non-responders had similar baseline characteristics so material difference between groups may be limited. Second, patients may have been more apt to report higher levels of satisfaction than they would using a more anonymous survey method, like a mailed paper and pencil or anonymous online survey. Speaking with an actual person may have resulted in patients feeling less inclined to score providers lower. Due to patient privacy restrictions for HCAHPS results, we were not able to compare patient satisfaction scores on the HCAHPS survey responders to those obtained via our third-party telephone survey company. Third, phone interviews were conducted in English and Spanish, effectively excluding groups of patients who spoke other languages. Fourth, the study used a large, continuous case series covering a full calendar year. It completely describes patients seen in the recent past but may not be generalizable to a broader time range. Because the dataset was large and complete, no power calculation was done. Finally, generalizing our results to different patient populations in different geographic locations should be done with caution.

## CONCLUSION

In summary, our study found several demographic factors significantly impacting satisfaction for our patients, including race/ethnicity, age, and patients on Medicare. As expected, longer wait times were associated with lower patient satisfaction. If reimbursement and ultimately physician compensation is impacted by patient satisfaction, institutions with higher proportions of certain demographic groups or that have shorter wait times may be at an advantage. Care factors, such as the number of laboratory or imaging tests ordered, were not associated with satisfaction; however, the number of medications administered during the visit and self-assessed improvement in the patient’s condition were associated with greater patient satisfaction. Future interventional studies might look at strategies that manage patient expectations regarding necessity of medications and when they should expect their condition to improve. Lower satisfaction among behavioral health patients is an area warranting further study.

## Figures and Tables

**Table 1:** Demographic, operational, and healthcare utilization characteristics across satisfaction scores.

	No rating	Score=1	Score=2	Score=3	Score=4	Score=5	Scored
All visits	14,330 (41%)	471 (2%)	558 (3%)	2,014 (10%)	5,347 (26%)	12,550 (60%)	20,940
Age group							
0–17 yrs	2,003 (42%)	52 (2%)	62 (2%)	252 (9%)	701 (25%)	1,739 (62%)	2,806
18–64 yrs	11,432 (41%)	377 (2%)	459 (3%)	1,620 (10%)	4,219 (26%)	9,528 (59%)	16,203
65 and older	895 (32%)	42 (2%)	37 (2%)	142 (7%)	427 (22%)	1,283 (66%)	1,931
Sex							
Female	7,092 (38%)	289 (3%)	328 (3%)	1,212 (11%)	2,873 (25%)	6,731 (59%)	11,433
Male	7,238 (43%)	182 (2%)	230 (2%)	802 (8%)	2,474 (26%)	5,819 (61%)	9,507
Race/ethnicity (5-level)							
White	7,030 (41%)	246 (2%)	294 (3%)	1,031 (10%)	2,824 (27%)	5,903 (57%)	10,298
Black	4,201 (39%)	144 (2%)	159 (2%)	625 (9%)	1,538 (23%)	4,160 (63%)	6,626
Hispanic	1,194 (39%)	25 (1%)	34 (2%)	118 (6%)	378 (20%)	1,294 (70%)	1,849
Other	1,135 (46%)	40 (3%)	42 (3%)	147 (11%)	388 (29%)	728 (54%)	1,345
Unknown	770 (48%)	16 (2%)	29 (4%)	93 (11%)	219 (27%)	465 (57%)	822
Emergency department language							
English	13,784 (41%)	467 (2%)	548 (3%)	1,968 (10%)	5,221 (26%)	11,918 (59%)	20,122
Spanish	493 (39%)	4 (1%)	8 (1%)	40 (5%)	110 (14%)	603 (79%)	765
Other	53 (50%)	0 (0%)	2 (4%)	6 (11%)	16 (30%)	29 (55%)	53
Zipcode median household income (2007–2011)							
$ 0–35,000	1,474 (41%)	45 (2%)	50 (2%)	208 (10%)	540 (25%)	1,313 (61%)	2,156
$35–50,000	5,861 (41%)	193 (2%)	231 (3%)	830 (10%)	2,129 (25%)	5,135 (60%)	8,518
$50–75,000	4,646 (40%)	154 (2%)	192 (3%)	672 (10%)	1,816 (26%)	4,175 (60%)	7,009
$75,000+	1,721 (41%)	69 (3%)	62 (2%)	230 (9%)	676 (27%)	1,480 (59%)	2,517
Unknown	628 (46%)	10 (1%)	23 (3%)	74 (10%)	186 (25%)	447 (60%)	740
ZIP code percent of households in poverty (2007–2011)							
0–10%	6,624 (40%)	235 (2%)	255 (3%)	925 (9%)	2,574 (26%)	5,857 (59%)	9,846
10–20%	3,799 (41%)	136 (2%)	150 (3%)	555 (10%)	1,424 (26%)	3,291 (59%)	5,556
20–30%	1,863 (41%)	47 (2%)	81 (3%)	255 (9%)	654 (24%)	1,682 (62%)	2,719
30%+	1,416 (41%)	43 (2%)	49 (2%)	205 (10%)	509 (24%)	1,273 (61%)	2,079
Unknown	628 (46%)	10 (1%)	23 (3%)	74 (10%)	186 (25%)	447 (60%)	740
Behavioral health treatment area							
Yes	711 (54%)	22 (4%)	14 (2%)	86 (14%)	183 (30%)	297 (49%)	602
No	13,619 (40%)	449 (2%)	544 (3%)	1,928 (9%)	5,164 (25%)	12,253 (60%)	20,338
Arrival means							
Private	9,738 (39%)	337 (2%)	420 (3%)	1,514 (10%)	3,968 (26%)	9,042 (59%)	15,281
Ambulance	2,808 (47%)	78 (2%)	79 (2%)	302 (9%)	780 (24%)	1,952 (61%)	3,191
Bus/walk	1,161 (43%)	37 (2%)	31 (2%)	117 (8%)	367 (24%)	997 (64%)	1,549
Other	623 (40%)	19 (2%)	28 (3%)	81 (9%)	232 (25%)	559 (61%)	919
Payer							
Medicaid	5,256 (40%)	210 (3%)	227 (3%)	793 (10%)	1,908 (24%)	4,877 (61%)	8,015
Commercial	4,466 (40%)	125 (2%)	184 (3%)	662 (10%)	2,005 (29%)	3,862 (56%)	6,838
Medicare	1,588 (34%)	87 (3%)	80 (3%)	272 (9%)	689 (22%)	1,985 (64%)	3,113
Self-pay	2,529 (51%)	41 (2%)	59 (2%)	231 (10%)	613 (25%)	1,484 (61%)	2,428
Other	491 (47%)	8 (1%)	8 (1%)	56 (10%)	132 (24%)	342 (63%)	546
Day of week							
Sunday	2,179 (40%)	70 (2%)	79 (2%)	274 (8%)	822 (25%)	1,998 (62%)	3,243
Monday	2,191 (42%)	72 (2%)	90 (3%)	308 (10%)	769 (26%)	1,760 (59%)	2,999
Tuesday	2,136 (41%)	78 (3%)	90 (3%)	305 (10%)	790 (26%)	1,794 (59%)	3,057
Wednesday	1,849 (39%)	59 (2%)	64 (2%)	276 (10%)	757 (26%)	1,710 (60%)	2,866
Thursday	1,974 (41%)	63 (2%)	76 (3%)	298 (10%)	712 (25%)	1,750 (60%)	2,899
Friday	1,883 (40%)	61 (2%)	83 (3%)	263 (9%)	724 (25%)	1,731 (60%)	2,862
Saturday	2,118 (41%)	68 (2%)	76 (3%)	290 (10%)	773 (26%)	1,807 (60%)	3,014
Weekend							
Yes	4,875 (40%)	151 (2%)	189 (3%)	664 (9%)	1,865 (26%)	4,336 (60%)	7,205
No	9,455 (41%)	320 (2%)	369 (3%)	1,350 (10%)	3,482 (25%)	8,214 (60%)	13,735
Night time (11pm–7am)							
Yes	3,528 (42%)	129 (3%)	151 (3%)	576 (12%)	1,233 (25%)	2,792 (57%)	4,881
No	10,802 (40%)	342 (2%)	407 (3%)	1,438 (9%)	4,114 (26%)	9,758 (61%)	16,059
Calendar month							
1	1,213 (41%)	30 (2%)	39 (2%)	155 (9%)	446 (26%)	1,044 (61%)	1,714
2	1,190 (48%)	29 (2%)	27 (2%)	112 (9%)	340 (26%)	799 (61%)	1,307
3	1,183 (41%)	30 (2%)	53 (3%)	143 (8%)	438 (26%)	1,047 (61%)	1,711
4	1,169 (41%)	57 (3%)	47 (3%)	154 (9%)	432 (25%)	1,017 (60%)	1,707
5	1,142 (39%)	49 (3%)	57 (3%)	219 (12%)	471 (27%)	958 (55%)	1,754
6	1,213 (40%)	48 (3%)	58 (3%)	198 (11%)	489 (27%)	1,043 (57%)	1,836
7	1,316 (40%)	35 (2%)	65 (3%)	197 (10%)	535 (28%)	1,103 (57%)	1,935
8	1,188 (38%)	47 (2%)	44 (2%)	171 (9%)	436 (22%)	1,260 (64%)	1,958
9	1,389 (47%)	30 (2%)	39 (2%)	148 (9%)	423 (27%)	943 (60%)	1,583
10	1,288 (41%)	37 (2%)	40 (2%)	169 (9%)	496 (27%)	1,074 (59%)	1,816
11	972 (34%)	37 (2%)	45 (2%)	166 (9%)	420 (23%)	1,182 (64%)	1,850
12	1,067 (38%)	42 (2%)	44 (2%)	182 (10%)	421 (24%)	1,080 (61%)	1,769
Calendar quarter							
1	3,586 (43%)	89 (2%)	119 (3%)	410 (9%)	1,224 (26%)	2,890 (61%)	5,824
2	3,524 (40%)	154 (3%)	162 (3%)	571 (11%)	1,392 (26%)	3,018 (57%)	6,487
3	3,893 (42%)	112 (2%)	148 (3%)	516 (9%)	1,394 (25%)	3,306 (60%)	6,627
4	3,327 (38%)	116 (2%)	129 (2%)	517 (10%)	1,337 (25%)	3,336 (61%)	6,681
Imaging done							
Yes	5,547 (38%)	176 (2%)	231 (3%)	811 (9%)	2,272 (26%)	5,393 (61%)	8,883
No	8,783 (42%)	295 (2%)	327 (3%)	1,203 (10%)	3,075 (26%)	7,157 (59%)	12,057
Labwork done							
Yes	5,221 (37%)	205 (2%)	226 (3%)	827 (9%)	2,215 (25%)	5,274 (60%)	8,747
No	9,109 (43%)	266 (2%)	332 (3%)	1,187 (10%)	3,132 (26%)	7,276 (60%)	12,193
Medications ordered in the emergency department							
Yes	8,558 (39%)	258 (2%)	339 (3%)	1,249 (10%)	3,325 (25%)	7,962 (61%)	13,133
No	5,772 (43%)	213 (3%)	219 (3%)	765 (10%)	2,022 (26%)	4,588 (59%)	7,807
Wait time: door to provider							
0–1 hour	8,751 (41%)	221 (2%)	234 (2%)	899 (7%)	2,989 (24%)	8,118 (65%)	12,461
1–2 hour	3,190 (39%)	105 (2%)	141 (3%)	546 (11%)	1,367 (27%)	2,818 (57%)	4,977
2–3 hour	1,395 (41%)	75 (4%)	83 (4%)	297 (15%)	595 (29%)	975 (48%)	2,025
3–4 hour	566 (40%)	30 (4%)	55 (7%)	153 (18%)	237 (28%)	371 (44%)	846
Unknown*	428 (40%)	40 (6%)	45 (7%)	119 (19%)	159 (25%)	268 (42%)	631
Wait time: provider to discharge							
0–1 hour	3,898 (43%)	99 (2%)	140 (3%)	462 (9%)	1,263 (24%)	3,214 (62%)	5,178
1–2 hour	3,916 (40%)	114 (2%)	140 (2%)	522 (9%)	1,477 (26%)	3,506 (61%)	5,759
2–3 hour	2,460 (38%)	87 (2%)	87 (2%)	387 (10%)	1,043 (26%)	2,436 (60%)	4,040
3–4 hour	1,511 (39%)	50 (2%)	54 (2%)	222 (9%)	621 (26%)	1,439 (60%)	2,386
4–5 hour	801 (39%)	26 (2%)	34 (3%)	124 (10%)	318 (25%)	749 (60%)	1,251
5–6 hour	489 (41%)	26 (4%)	17 (2%)	75 (11%)	201 (28%)	387 (55%)	706
6–7 hour	248 (39%)	11 (3%)	12 (3%)	49 (13%)	94 (24%)	218 (57%)	384
7+ hour	155 (43%)	5 (2%)	8 (4%)	24 (12%)	57 (28%)	109 (54%)	203
Unknown*	852 (45%)	53 (5%)	66 (6%)	149 (14%)	273 (26%)	492 (48%)	1,033
Self-reported medical condition change							
Improved	399 (3%)	197 (1%)	292 (2%)	1,243 (8%)	3,837 (25%)	9,514 (63%)	15,083
Same	171 (3%)	177 (4%)	198 (4%)	625 (13%)	1,282 (27%)	2,533 (53%)	4,815
Worsened	79 (7%)	96 (9%)	68 (7%)	145 (14%)	226 (22%)	497 (48%)	1,032
(Unknown)	13,681 (100%)	1 (10%)	0 (0%)	1 (10%)	2 (20%)	6 (60%)	10

Percent for no rating is based on all patients: percent for a given satisfaction score is based on all patients with a score.

* Unknown due to missing timestamp data or timestamp data resulting in a negative wait time.

**Table 2:** Univariate predictors of a one-unit increase in patient satisfaction score.

Predictor	OR (95% CI)	p-value
Age (years)		
<65	Ref	
≥65	1.36 (1.23, 1.49)	<0.001*
Sex		
Male	Ref	
Female	0.88 (0.83, 0.93)	<0.001*
Race		
Non-Hispanic White or other	Ref	
Non-Hispanic Black	1.25 (1.18, 1.32)	<0.001*
Hispanic	1.75 (1.58, 1.95)	<0.001*
10% increase in poverty		
n/a	1.03 (1.00, 1.06)	0.06
Primary language		
English or other	Ref	
Spanish	2.55 (2.15, 3.03)	<0.001*
Mode of arrival		
Private	Ref	
Ambulance	1.08 (1.00, 1.16)	<0.001*
Bus/walk	1.25 (1.12, 1.39)	<0.001*
Other	1.08 (0.94, 1.23)	<0.001*
Payor		
Commercial	Ref	
Medicaid	1.13 (1.06, 1.20)	<0.001*
Medicare	1.27 (1.17, 1.38)	<0.001*
Other	1.23 (1.03, 1.46)	<0.001*
Self-pay	1.17 (1.07, 1.29)	<0.001*
Day of the week		
Weekday	Ref	0.35
Weekend	1.03 (0.97, 1.09)	
Shift		
Non-night	Ref	
Night (11pm–7am)	0.84 (0.79, 0.89)	<0.001*
Wait time: door to doctor (hour)		
n/a	0.70 (0.67, 0.72)	<0.001*
Wait time: doctor to disposition (hour)		
n/a	0.96 (0.94, 0.98)	<0.001*
Treatment location		
Non-behavioral health	Ref	
Behavioral health	0.65 (0.56, 0.76)	<0.001*
Medication ordered prior to discharge		
No	Ref	
Yes	1.09 (1.03, 1.15)	0.002*
Self-assessed medical condition		
Better	Ref	
Unchanged	0.60 (0.57, 0.64)	<0.001*
Worsened	0.43 (0.38, 0.48)	<0.001*

* Represents statistically significant figures.

**Table 3:** Multivariate analysis of a one-unit increase in patient satisfaction score.

Predictor	OR (95% CI)	p-value
Race and language		
Non-Hispanic White and all other	Ref	
Non-Hispanic Black	1.25 (1.17, 1.33)	<0.001*
English-speaking Hispanic	1.34 (1.17, 1.52)	<0.001*
Spanish-speaking Hispanic	3.30 (2.73, 3.99)	<0.001*
Mode of arrival		
All other	Ref	
Bus/walk	1.22 (1.09, 1.36)	<0.001*
Age and payor		
Age <65, not on Medicare	Ref	
Age ≥65, on Medicare	1.54 (1.39, 1.71)	<0.0001*
Age <65, on Medicare	1.14 (1.01, 1.27)	<0.0001*
Treatment location		
Non-Behavioral health	Ref	
Behavioral health	0.65 (0.55, 0.78)	<0.0001*
Wait time: door to doctor		
Per hour increase	0.67 (0.64, 0.69)	<0.0001*
Wait time: doctor to disposition		
Per hour increase	0.92 (0.91, 0.94)	<0.0001*
Medication ordered prior to discharge		
No	Ref	
Yes	1.12 (1.05, 1.18)	<0.001*
Self-assessed medical condition		
Better	Ref	
Unchanged	0.60 (0.56, 0.64)	<0.0001*
Worsened	0.39 (0.35, 0.44)	<0.0001*

* Represents statistically significant figures.
